# Forecasting of Typhoon-Induced Wind-Wave by Using Convolutional Deep Learning on Fused Data of Remote Sensing and Ground Measurements

**DOI:** 10.3390/s21155234

**Published:** 2021-08-02

**Authors:** Chih-Chiang Wei, Hao-Chun Chang

**Affiliations:** Department of Marine Environmental Informatics & Center of Excellence for Ocean Engineering, National Taiwan Ocean University, Keelung 20224, Taiwan; 10881006@mail.ntou.edu.tw

**Keywords:** deep learning, radar mosaics, typhoon, wind speed, wave height, neural networks

## Abstract

Taiwan is an island, and its economic activities are primarily dependent on maritime transport and international trade. However, Taiwan is also located in the region of typhoon development in the Northwestern Pacific Basin. Thus, it frequently receives strong winds and large waves brought by typhoons, which pose a considerable threat to port operations. To determine the real-time status of winds and waves brought by typhoons near the coasts of major ports in Taiwan, this study developed models for predicting the wind speed and wave height near the coasts of ports during typhoon periods. The forecasting horizons range from 1 to 6 h. In this study, the gated recurrent unit (GRU) neural networks and convolutional neural networks (CNNs) were combined and adopted to formulate the typhoon-induced wind and wave height prediction models. This work designed two wind speed prediction models (WIND-1 and WIND-2) and four wave height prediction models (WAVE-1 to WAVE-4), which are based on the WIND-1 and WIND-2 model outcomes. The Longdong and Liuqiu Buoys were the experiment locations. The observatory data from the ground stations and buoys, as well as radar reflectivity images, were adopted. The results indicated that, first, WIND-2 has a superior wind speed prediction performance to WIND-1, where WIND-2 can be used to identify the temporal and spatial changes in wind speeds using ground station data and reflectivity images. Second, WAVE-4 has the optimal wave height prediction performance, followed by WAVE-3, WAVE-2, and WAVE-1. The results of WAVE-4 revealed using the designed models with in-situ and reflectivity data directly yielded optimal predictions of the wind-based wave heights. Overall, the results indicated that the presented combination models were able to extract the spatial image features using multiple convolutional and pooling layers and provide useful information from time-series data using the GRU memory cell units. Overall, the presented models could exhibit promising results.

## 1. Introduction

**Figure 1:**
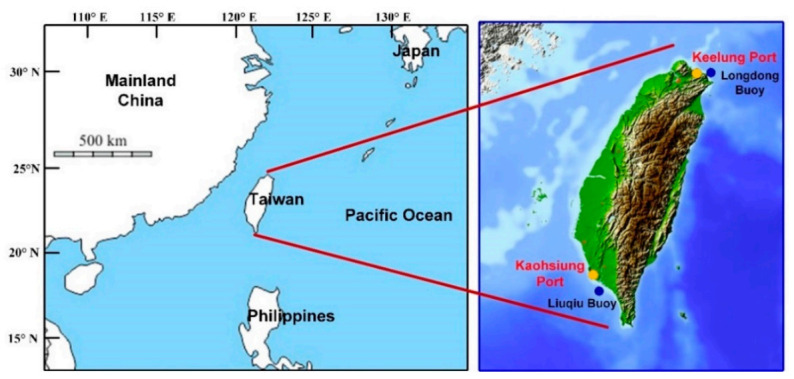
Locations of Taiwan, Keelung Port, and Kaohsiung Port.

Taiwan, located at 120°–122° E and 22°–25° N, experiences subtropical weather, is an island with over 95% of its commodity trade being conducted through sea transport (TIPC 2020), and boasts two major international ports (Figure 1)—namely, Keelung and Kaohsiung Ports. Keelung Port is located on the north coast of Taiwan, with the Northeast Asia shipping route passing through the port. Kaohsiung Port is located on the southwest coast of Taiwan. This port is the largest port in Taiwan and the 15th largest container port worldwide. The two ports are crucial for maritime transport. However, Taiwan is located in the typhoon development area of the Northwestern Pacific Basin and, thus, is often struck by typhoons. Moreover, Taiwan is influenced by monsoons and the oceanic climate and often experiences severe weather. Such weather has a considerable influence on port operations.

Typhoons are strong tropical cyclones with high destructive power that often form in the summer and autumn. The heavy rain, strong winds, and large waves brought by typhoons influence ship safety and the docking operation at ports. In addition, typhoons damage facilities inside and outside ports [1]. For example, Typhoon Meranti resulted in the breaking of a container ship cable at Kaohsiung Port on 14 September 2016, which resulted in a loss of USD$40 million in equipment. Typhoon Nesat caused a cruise ship cable to break on 29 July 2017, and the cruise ship subsequently collided with multiple vessels. To reduce the threat to port safety at times of typhoons, predicting the winds and waves during typhoons is crucial. The goal of this study was to predict instant changes in wind speed and wave height over several hours in the water near the coasts of the international ports in Taiwan during typhoons. The wind and wave information obtained can subsequently improve ship navigation safety and port operation efficiency.

Currently, four weather surveillance radars of the Central Weather Bureau (CWB) are in operation. These radars are located at Wufenshan, Hualien, Cigu, and Kenting. The overall scan range of these four radars spans Taiwan and the neighboring waters, including the Pacific Ocean, East China Sea, Taiwan Strait, and Luzon Strait. These radars constitute the weather radar observation network in Taiwan [2]. Weather radar reflectivities are electromagnetic signals reflected by precipitation particles (rain, snow, and hail) in the atmosphere. Weather radar data have the features of being observable 24 h a day, high spatial coverage density, high temporal resolution, and instant data access. Therefore, these data can be used to identify the spatial reflectivity changes over a large area and the characteristics of temporal changes in air currents.

Research on weather radar data dates back to the use of Doppler radar data by reference [3] to estimate the radial wind field. Lhermitte and Atlas [4] proposed the velocity–azimuth display (VAD) method, which involves using Doppler radar to measure the wind field speed, wind direction, and precipitation falling speed for large-scale precipitation. Browning and Wexler [5] used a single Doppler radar to explore the movement of precipitation particles on the basis of the VAD method. This method is used to measure the wind field movement in large-scale precipitation. Marks and Houze [6,7] used the observatory data of airplane radars to investigate the horizontal radial wind speed and vertical air movement of the wind field structure around typhoon centers. Lee et al. [8,9] used the ground-based velocity track display technique with a single ground Doppler radar to examine the average tangential wind speed and average radial wind speed of the internal circulation structure of typhoons. However, due to the insufficient information provided by a single Doppler radar, as well as the influences of terrain and the distance between the typhoon center and the radar on wind field retrieval, wind field retrieval techniques based on the use of a dual-Doppler radar have been developed [10,11,12]. Park and Lee [13] developed a wind field retrieval technique based on the use of multiple Doppler radars. These studies have confirmed the correlations between radar reflectivities and wind field changes. Thus, the temporal and spatial features of wind fields can be feasibly extracted from radar reflectivity images.

In general, wind and wave estimation and forecasting are made on the basis of numerical models, which often yield accurate and physically-based results. Examples of such models are numerical weather prediction models (e.g., MM5 [14] and WRF [15,16,17]) and ocean wave models (e.g., WAVEWATCH series [18] and SWAN [19]). However, developing such a forecasting system requires various boundary data and initial data and is highly mathematically complicated.

With the development of artificial intelligence, numerous machine learning algorithms, such as decision trees, support vector machines, and deep neural networks, such as multilayer perceptrons (MLPs) and convolutional neural networks (CNNs), have been applied to the field of natural science. The advantages of machine learning methods are that they effectively identify and learn correlated patterns between input datasets and the corresponding target values [20,21]. However, they are less subject to the constraints of physical mechanisms [22,23]. Some studies have conducted wind speed and wave height predictions by using machine learning models [24,25,26,27]. For example, Wang et al. [28] proposed a wind speed prediction method that involved the use of a wavelet transform, deep belief networks, and quantile regressions based on deep learning. Wei et al. [29] used the backpropagation neural network algorithm to construct a wind speed prediction model. This model was used to assess the prediction precision of typhoon routes for different locations and periods. Mandal and Prabaharan [30] used recurrent neural networks to construct a wave prediction model. Zhang and Dai [31] developed a conditional restricted Boltzmann machine in a classical deep belief network by using one-dimensional (1D) data obtained from buoys to predict the wave height. Wei and Cheng [32] proposed a model based on deep recurrent neural networks for predicting the wind speed and wave height during typhoons. They performed the predictions by using 1D ground observatory data. Most of these studies adopted the data provided by ground stations. Few studies have used CNNs to extract the wind field features of radar reflectivity images.

CNNs are used for image recognition, natural language processing, and video analysis. LeCun [33] proposed the prototype of CNNs, as well as the concepts of weight sharing and feature maps. LeNet (LeCun et al. [34]) is the first CNN with a complete structure. It comprises a convolutional layer, pooling layer, fully connected layer, and nonlinear activation function. Krizhevsky et al. [35] proposed the AlexNet model to fix some of the defects of CNNs, such as the max pooling method that was adopted to avoid missing features, and the dropout method was introduced to prevent overfitting. Since AlexNet, various CNN models, such as VGGNet (Simonyan and Zisserman [36]), GoogLeNet (Szegedy et al. [37]), and ResNet (He et al. [38]), have been proposed. VGGNet is a classic CNN model. It stacks CNNs through a small convolution process, which makes the model deeper. GoogLeNet and ResNet are network structures based on VGGNet.

This study referred to the most commonly used CNN models, such as VGG16, and designed variations of the VGGNet architectures for formulating wind–wave prediction models using the image data from radar reflectivity. Moreover, the ground station data and buoy data were added to involve the in-situ observation information. In general, a limitation of the MLP architecture is that it assumes that all inputs and outputs are independent of each other [39]. In order for an MLP to formulate a time-series forecast problem, it is necessary to include some temporal information in the input data. One machine learning approach used for time-series forecasts is the long short-term memory (LSTM) neural network [40], which is a type of recurrent neural network (RNN) that can learn the temporal dynamics of sequential data and address the vanishing gradient problems in RNNs [41]. Although LSTM has made an increase in the accuracy in numerous fields, e.g., weather forecasting, it also takes a longer time to train a model [42]. Recently, the gated recurrent unit (GRU) neural network [43,44], a variant of the LSTM. has fewer parameters than the LSTM and accelerates the training and equivalent capability [45]. The difference between LSTM and GRU is that LSTM has three gates (i.e., input, output, and forget gates), whereas GRU has two gates (i.e., reset and update gates). The GRU controls the flow of information like the LSTM unit but without having to use a memory unit [46]. As a result, GRU not only inherits the merits of the LSTM network but also greatly shortens the training time [39,47]. Accordingly, this study employed a GRU neural network to learn the temporal dynamics of time sequential data to accelerate the model computing time.

In this study, RNNs and CNNs were combined and then used as the modeling algorithms. This combination of network architectures is able to capture time dependencies on the features extracted by convolutional operations [48,49,50,51]. This hybrid model has been applied in weather and hydrological prediction problems; for example, Zhu et al. [52] and Shivam et al. [53] transformed wind speed data into wind speed intensity images and used CNNs to capture the temporal and spatial features of wind fields. Moishin et al. [54] built a hybrid deep learning algorithm integrating the predictive merits of CNN and the LSTM network to forecast the future occurrence of flood events.

In conclusion, the major contributions of this study are as follows. This paper designed CNNs combined with RNN-based GRUs models that enable us to fuse these 1D observation data and 2D radar reflectivity image data for addressing the extraction of spatial features using convolutional operations and time consistency using recurrent operations. In contrast, past studies (such as references [29,32]) proposed a model based on RNNs for predicting the wind velocity and wave height, which performed the predictions only using 1D ground observatory data. Thus, they did not address the issue regrading fusing the 1D in-situ observations and 2D spatial image data. Therefore, those models cannot extract the temporal and spatial features from the 1D and 2D time-series data. Specifically, the advanced machine learning techniques were performed in the work; that is, the CNNs combined with GRUs were used to extract the temporal and spatial features of the wind and waves from those in-situ ground data and radar reflectivity images.

## 2. Data Sources

The study area comprises the coastal waters of Keelung and Kaohsiung Ports. According to the CWB of Taiwan, Longdong Buoy is located in the coastal waters of Keelung Port, and Liuqiu Buoy is located in the coastal waters of Kaohsiung Port (Figure 1). The data of 21 typhoons that occurred between 2013 and 2019 were collected in this study from the Typhoon Database of the CWB [55]. The typhoon data were collected starting from 2013, because the resolution and color appearance of the radar reflectivity images were different from those of the radar images captured before 2013. Table 1 presents the data of 5 severe, 9 moderate, and 7 mild typhoons. According to the CWB, mild, moderate, and severe typhoons are defined as those having maximum wind speeds of 17.2–32.6, 32.7–50.9, and >51 m/s, respectively.

**Figure 2:**
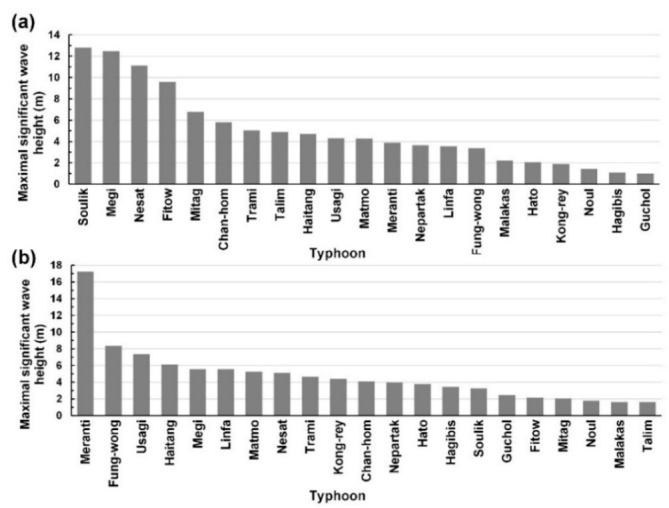
Maximal significant wave height for the typhoons at (**a**) Longdong Buoy and (**b**) Liuqiu Buoy.

Hourly observatory data from ground stations and buoys, as well as radar reflectivity images, were collected for the typhoons presented in Table 1. Data were collected from the ground stations: Keelung Station and Kaohsiung Station. Data on the following attributes were collected for the both stations: air pressure on the ground (P_air_; hPa), air pressure at sea level (P_asl_ G_2_; hPa), surface temperature (T_sfc_; °C), surface dew point temperature (T_sdp_; °C), surface relative humidity (RH_sfr_; %), surface vapor pressure (P_sfv_; hPa), average surface wind speed (WS_avs_; m/s), average surface wind direction (WD_avs_; degree), maximum 10-min mean surface wind speed (WS_msf_; m/s), maximum 10-min mean surface wind direction (WD_msf_; °), instantaneous maximum surface wind speed (WS_inm_; m/s), instantaneous maximum surface wind direction (WD_inm_; °), hourly ground precipitation (R_rlg_; mm/h), and rainfall duration within 1 h (D_rnf_; h). In this study, these attributes were defined as the ground meteorological dataset {**G**}. In addition, information on the following marine attributes was collected from Longdong Buoy and Liuqiu Buoy: average wind speed on the sea (V; m/s; defined as the marine wind dataset {**V**}) and significant wave height (H; m; defined as the marine wave height dataset {**H**}). Figure 2 indicates that the maximal significant wave height caused by the typhoons at Longdong Buoy and Liuqiu Buoy were different.

For all the radar reflectivity images collected in this study (defined as the radar reflectivity images {**I**}), the spatial resolution was 1024 × 1024 pixels. One pixel corresponds to an actual distance of 0.7 × 0.7 km. The CWB of Taiwan has four S-band (10-cm wavelength) weather surveillance radar (WSR) systems; this type of radar is also referred to as WSR-88D (Weather Surveillance Radar—1988 Doppler) [56]. The radar product of CWB uses the skill of a constant altitude plan position indicator (CAPPI). A CAPPI is calculated and interpolated at different elevations (e.g., at scanning angles of 0.5°, 1.4°, 2.4°, 3.4°, 4.3°, 6.0°, 9.9°, 14.6°, and 19.5° at Hualien Radar). According to reference [57], the CAPPI is at the height of 4 km for the radar product of CWB. Although the 6-min volume scanning radar mosaic are produced, it is inconsistent with the 1-h sampling frequency of the in-situ data obtained from the ground stations and buoys. Therefore, this study decided that the radar products were collected one sample per hour.

## 3. Model Development

In this study, deep-learning algorithms were used to construct wind and wave prediction models. During the wind and wave prediction process, the radar reflectivity images were combined with the data obtained from ground stations and buoys during typhoon periods. The wind and wave models used in this study were constructed in two stages. First, wind speed prediction models were used to predict the wind speeds during the typhoon period. Then, the wind speed prediction results were input to wave height prediction models for wave height prediction. Moreover, we added a one-stage model case, which directly predicts wave heights without wind speed estimations.

### 3.1. Model Cases

To deal with the various data, i.e., 1D in-situ observations and two-dimensional (2D) images, this study designed models using convolutional deep learning to integrate the 1D and 2D data. The designed models were the combinations of the CNNs (using convolutional layers) and GRUs (using GRU layers) and used to extract the temporal and spatial features of the wind and wave from those 1D and 2D data. In addition, the combination of the CNNs and MLPs (using dense layers) was used as a benchmark model. In summary, Table 2 lists all the designed model cases for wind speed (i.e., WIND-1 and WIND-2) and wave height (i.e., WAVE-1 to WAVE-4) and their corresponding algorithms and data used. Here, our proposed cases were WIND-2, WAVE-3, and WAVE-4, and the other cases using past studies were WIND-1 [21,23,29], WAVE-1 [32], and WAVE-2 [32]. The network architectures of these model cases were described in the following sections.

#### 3.1.1. WIND-1 and WIND-2 Models

**Figure 3:**
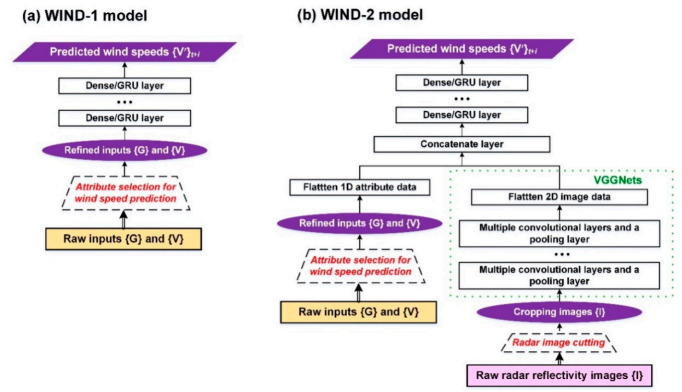
Schematics of the wind prediction models: (**a**) WIND-1 and (**b**) WIND-2.

Two models—namely, WIND-1 and WIND-2—were constructed for wind speed prediction in this study (Figure 3). The forecasting horizons ranged from 1 to 6 h. Therefore, the target in WIND-1 and WIND-2 was the wind speeds of the buoy at (*t* + *i*)*_i_*_=1,6_. The details of the WIND-based models were as follows.

- WIND-1 model

The meteorological dataset {**G**} and marine wind dataset {**V**} were used as the input data for WIND-1 (Figure 3a). A dense/GRU layer was adopted for constructing WIND-1 (the settings of the model parameters; see Section 3.3.1). After the parameter training process of stacked recurrent layers, the prediction results {**V**’} were obtained. For both WIND-based models (i.e., WIND-1 and WIND-2), the attribute selection method, i.e., the correlation-based criterion method, was used to select attributes from the {**G**} and {**V**} datasets useful for wind speed prediction and set them as the input data. In addition, a traditional linear regression-based method was applied to the WIND-1 model (using the same inputs and model targets) and tested as a benchmark model (namely, WIND-1R).

- WIND-2 model

**Figure 4:**
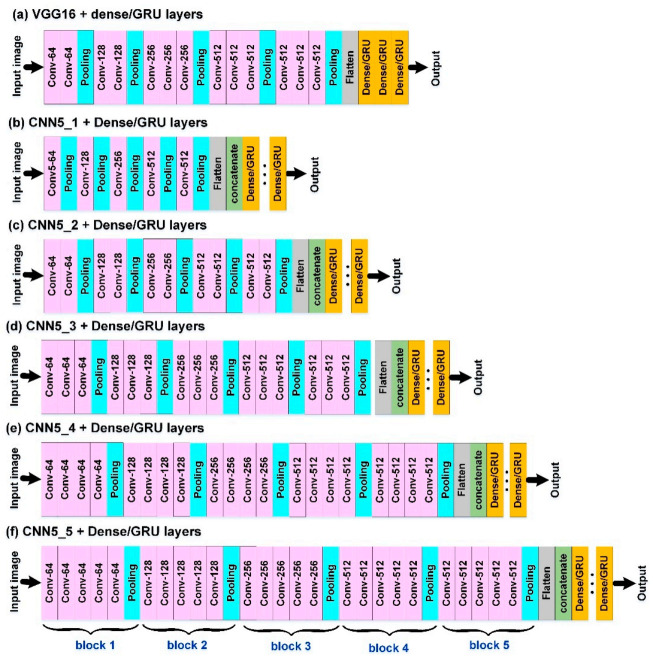
Architectures of the VGGNets of (**a**) classical VGG16 and the variations of (**b**) CNN5_1, (**c**) CNN5_2, (**d**) CNN5_3, (**e**) CNN5_4, and (**f**) CNN5_5 (in the figure, the input image size is 64 × 64 pixels as an example).

The datasets of {**G**} and {**V**}, as well as the reflectivity images {**I**}, were used as the input data for WIND-2. Due to the requirement of processing 2D images, a CNN algorithm combined with a dense/GRU algorithm was adopted to construct a WIND-2 model (Figure 3b). For feature extraction from the images {**I**}, the VGGNet network structure proposed by reference [36] is referred to in this study. The features are extracted from VGG16, which consists of 13 convolution layers and three fully connected layers. However, the fixed architectures of VGG16 cannot guarantee suitable networks for the studied data. To identify the optimal network structures, we designed varied architectures that were similar to VGG16 (namely, VGGNet variations). Figure 4a is the typical VGG16 architecture where the input image size (an example of 64 × 64 pixels) is passed through a stack of convolutional layers and max pooling layers. The developed VGGNet variations of CNN5_1, CNN5_2, CNN5_3, CNN5_4, and CNN5_5 (Figure 4b–f) are modified by using the architecture of VGG16. That is, the network structure in each of VGGNet variations comprises five blocks of multiple convolutional layers and a pooling layer. CNN5_4, for example, represents the structure with five blocks and four convolutional layers in each block. The VGGNets (including VGG16 and its variations) were formulated in the study. After feature extraction, effective feature maps can be obtained for model training [58]. For the settings of the model parameters, see Section 3.3.2.

Subsequently, to integrate the 1D datasets {**G**} and {**V**} and 2D imagery {**I**}, the flatten layer is first used to flatten the 1D and 2D data. Then, the concatenate layer is used to combine the data into 1D arrays. Finally, the parameter training of dense/GRU layers is conducted to obtain the wind speed predictions {**V**’}. To process the imagery {**I**}, the original bitmaps are input into the WIND-2 model, and the original radar reflectivity images are then cropped. Subsequently, the inputs were obtained from the different sizes of the cropped reflectivity images. The cropping method involves using the buoy location as the center and cropping the images outward into different sizes. Then, the most suitable image sizes are identified for a specific station when a typhoon passes (for details of the process, see Section 3.3.2).

#### 3.1.2. WAVE-1 to WAVE-4 Models

**Figure 5:**
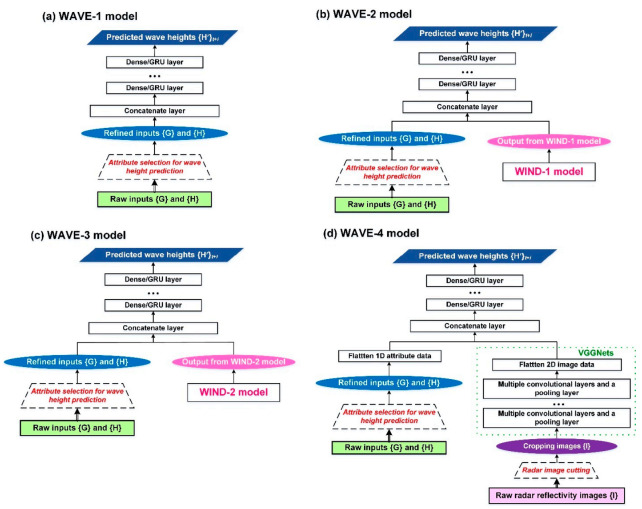
Schematics of the wave height prediction models: (**a**) WAVE-1, (**b**) WAVE-2, (**c**) WAVE-3, and (**d**) WAVE-4.

Four wave prediction models—namely, WAVE-1, WAVE-2, WAVE-3, and WAVE-4—were also constructed (Figure 5). Here, the targets of these WAVE-based models are the wave heights of a buoy at (*t* + *i*)*_i_*_=1,6_. The details of WAVE-based models were described as follows.

- WAVE-1 model

The meteorological dataset {**G**} and wave height dataset {**H**} were used as the input data for WAVE-1 (Figure 5a). An RNN algorithm was adopted for constructing the WAVE-1 model. For all WAVE-based models, the attribute selection method was used to select the attributes from the {**G**} and {H} datasets useful for wave height predictions and set them as the input data. The parameter training of the stacked recurrent layers was conducted to obtain the wave height predictions {**H**’}.

- WAVE-2 model using WIND-1 model outcomes

For the WAVE-2 model (Figure 5b), the wind speed prediction results {**V**’} are obtained using WIND-1. These results are then fused with the {**G**}, {**H**}, and {**V**’} datasets by using the concatenate layer, and parameter training of the stacked recurrent layers is conducted to obtain the wave height predictions {**H**’}.

- WAVE-3 model using WIND-2 model outcomes

For WAVE-3 (Figure 5c), the datasets of {**G**} and {**H**}, as well as the wind speed prediction results {**V**’} of WIND-2, were used as the input data. As in WAVE-2, the concatenate layer is used to fuse these data into 1D arrays, and parameter training is conducted to obtain the {**H**’}.

- WAVE-4 model

The WAVE-4 prediction model conducts one-stage wave height predictions (Figure 5d). The datasets of {**G**} and {**H**}, as well as images {**I**}, were used as the input data for WAVE-4. Similarly, the VGGNets were applied and verified the suitable network structures in the WAVE-4 model. In addition, the original reflectivity images were cropped, and the optimal image sizes were determined for the wave height prediction (see Section 3.4).

### 3.2. Typhoon Data Division

Prior to modeling, the datasets were divided into three datasets. Three typhoons—namely, Typhoons Usagi, Fung-wong, and Megi—had the respective intensities of severe, mild, and moderate and were selected to comprise the testing set for modeling. Typhoons Haitang, Nesat, Nepartak, and Mitag (with the intensities of mild, moderate, and severe, respectively) were used for the validation set. The other 14 typhoons were placed in the training set (including typhoons Soulik and Meranti, which had the maximal wave heights respectively at the Longdong Buoy and Liuqiu Buoy). In total, the training, validation, and testing sets comprised 1056, 288, and 288 hourly records, respectively.

**Figure 6:**
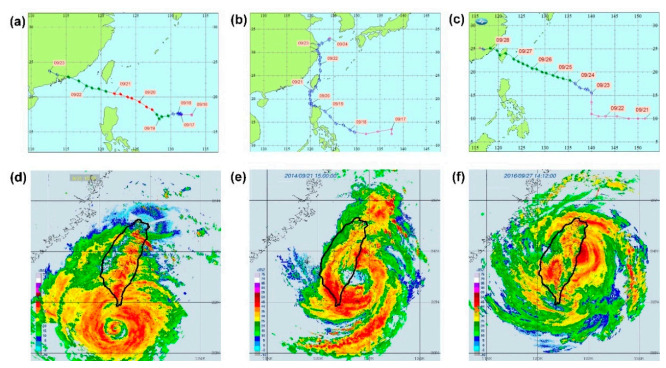
Paths of (**a**) Typhoon Usagi in 2013, (**b**) Typhoon Fung-wong in 2014, and (**c**) Typhoon Megi in 2016, as well as the radar reflectivity imagery of (**d**) Typhoon Usagi at 15:00 local standard time (LST) on 21 September 2013, (**e**) Typhoon Fung-wong at 15:00 LST on 21 September 2014, and (**f**) Typhoon Megi at 14:00 LST on 27 September 2016.

Figure 6 shows the radar reflectivity images of the routes of the testing set (typhoons Usagi, Fung-wong, and Megi) when they landed or were closest to landing. Figure 6a,d reveals that Typhoon Usagi moved westward through the Luzon Strait, which is the strait between Taiwan and Luzon Island of the Philippines. Figure 6b,e indicates that Typhoon Fung-wong passed through the east coast of Taiwan moving from south to north. For Typhoons Usagi and Fung-wong, large reflectivity dBZ values (decibels relative to the reflectivity factor (Z)) and extremely high significant wave heights were observed in the areas near Liuqiu Buoy. The unit dBZ is a logarithmic dimensionless unit used in radar imaging. Moreover, Figure 6c,f indicates that Typhoon Megi landed on the northeastern coast of Taiwan and that large dBZ values were observed in the areas near Longdong Buoy in the radar reflectivity images. These areas also had extremely large significant wave heights.

### 3.3. Modeling for Wind Predictions

The developed models were implemented using the open-source scikit-learn and Keras libraries in Python 3.7 (Python Software Foundation, Wilmington, DE, USA [59]).

#### 3.3.1. Construction of WIND-1 Model

To construct a WIND-1 model, the settings of the dense/GRU layers were as follows: activation function = rectified linear units (ReLU), the dropout rate of the dropout layer = 0.25, loss function = mean squared errors, metrics = root mean squared errors (RMSE), the epoch sizes = 500, and optimizer = adaptive moment estimation (Adam). The min–max normalization was used to rescale the range of features in (0, 1). The Adam optimizer was used to learn the learning rate. Adam is a first-order optimization algorithm that can replace the traditional stochastic gradient descent algorithm, and it can adapt the learning rate to the parameters [60]. The initial learning rate was set to 0.01. The hyperparameters of the number of dense/GRU layers and the number of neurons in a dense/GRU layer were calibrated. The optimal parameter combinations with the minimal RMSEs were searched. The RMSE is defined as follows:

(1) $$\mathrm{RMSE}=\sqrt{\frac{\sum_{i=1}^{N_{k}}{\left(y_{k,i}^{\mathrm{obs}}-{\hat{y}}_{k,i}^{\mathrm{pre}}\right)}^{2}}{N_{k}}}$$

where *N_k_* is the total number of data points (i.e., the records of wind speed and wave height) at buoy *k*, and $y_{k,i}^{\mathrm{obs}}$ and ${\hat{y}}_{k,i}^{\mathrm{pre}}$ are the observation and prediction values for the *i*th data point at buoy *k*, respectively. The effect of each error on the RMSE is proportional to the size of the squared error. It should be noted that it is influenced heavily by large errors compared to smaller errors [61].

The calibration range for the number of dense/GRU layers was 1–8, and the calibration range for the number of neurons in a dense/GRU layer was 20–260. Table 3 presents the calibration results of the optimal parameters for a validation set using all the possible combinations of these parameters.

#### 3.3.2. Construction of WIND-2 Model

**Figure 7:**
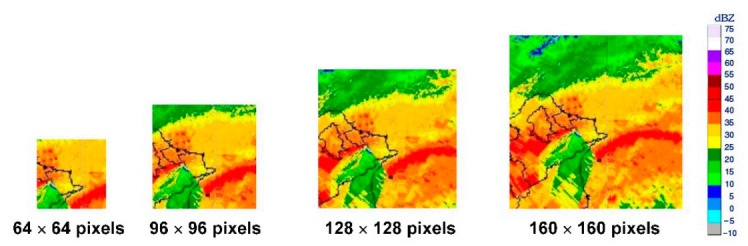
Cropped radar reflectivity images for Typhoon Megi.

When constructing the WIND-2 models, the pooling layers were passed five blocks through the architectures the VGGNet variations (Figure 4b–f). For the input images of the VGGNet variations, each time the pooling layers were passed through, the original images were shrunk to half their size. After five passes, for example, using CNN_5_4 architecture, the images were shrunk to 1/32nd their original size. Consequently, 32 pixels were used as the unit to crop the radar reflectivity images for the subsequent model calculations. The cropped images of four sizes: 64 × 64, 96 × 96, 128 × 128, and 160 × 160 pixels were used in this study. The original radar reflectivity images (1024 × 1024 pixels) were cropped into squares with the buoys as the center. The upper-left corners of the images were used as the origin to measure the locations of the buoys. The pixel location of the Longdong Buoy was x = 630 and y = 286. The pixel location of the Liuqiu Buoy was x = 420 and y = 661. The locations of the two buoys were used as centers to crop the images into different sizes. Figure 7, for example, depicts the spatial ranges covered by the four image sizes when Longdong Buoy was used as the center for the image cropping of 64, 96, 128, and 160 pixels for Typhoon Megi.

The cropped images were labeled when used as model inputs of VGGNet variations. According to the legend of dBZ (Figure 7), there were 17 colors (where the dBZ ranged from −10 to 75 dBZ, divided by 5 dBZ). Therefore, the number of categories was 17. These cropped images were then encoded into RGB channels (i.e., red, green, and blue), and pixel values at each channel were integer values between 0 and 255. The pixel-based RGB representation of an image was then used as the model input.

When running the VGGNets for the WIND-2 model, the parameter settings of the convolutional and pooling layers were as follows: kernel size = (5, 5), padding method = same, max pooling with pool size = (2, 2), the activation function = ReLU, and the dropout rate of the dropout layer = 0.25. The number of filters = 64, 128, 256, 512, and 512, respectively, in blocks 1–5 of the multiple convolutional layers. After flattening the 1D attributes and 2D feature maps, both flattened arrays were combined by using a concatenate layer. Subsequently, the dense/GRU layer environmental setups (i.e., activation function, dropout rate, loss function, metrics, and optimizer) are the same as in the WIND-1 model. The epoch sizes in WIND-2 are the epoch sizes = 300.

The structure types of VGGNets; the cropped image sizes (64, 96, 128, and 160 pixels); the number of dense/GRU layers (a range of 1–5); and the number of neurons in a dense/GRU layer (a range of 1000–3500) were calibrated. Table 4 reveals the optimal parameter combinations for Longdong Buoy and Liuqiu Buoy.

### 3.4. Modeling for Wave Predictions

This section describes the modeling conducted for the wave prediction. First, attributes with moderate or strong correlations were selected as the wave height attributes. Thus, 10 attributes were selected for the Longdong Buoy (P_air_, P_asl_, T_sfc_, T_sdp_, P_sfv_, WS_avs_, WS_msf_, WS_inm_, D_rnf_, and H) (with the corresponding correlation coefficients = 0.384, 0.385, −0.488, −0.474, −0.476, 0.577, 0.605, 0.636, 0.403, and 1), and 6 attributes were selected from Liuqiu Buoy (P_air_, P_asl_, WS_msf_, WS_inm_, D_rnf_, and H) (with the corresponding correlation coefficients = −0.346, −0.346, 0.319, 0.377, 0.333, and 1).

The wave height prediction models were constructed according to WAVE-1 and WAVE-4. The model environmental setups of WAVE-1, WAVE-2, and WAVE-3 were the same as in WIND-1. A trial-and-error method was used to find the optimal hyperparameter combinations. The calibration range for the number of dense/GRU layers was 1–8, and the calibration range for the number of neurons in a dense/GRU layer was 20–260. Table 5 presents the calibration results of the optimal parameters using a validation set for both buoys.

Moreover, the model setups of WAVE-4 were the same as in WIND-2. The structure types of the VGGNets, the cropped image sizes, the number of dense/GRU layers, and the number of neurons in a dense/GRU layer were determined. The calibration range for the cropped image sizes = 64, 96, 128, and 160 pixels; the number of dense/GRU layers (a range of 1–5); and the number of neurons in a dense/GRU layer (a range of 1000–3500) were calibrated. Table 6 presents the optimal parameter results regarding the structure types, the cropped image sizes, the number of dense/GRU layers, and the number of neurons in a dense/GRU layer for both buoys.

**Figure A1:**
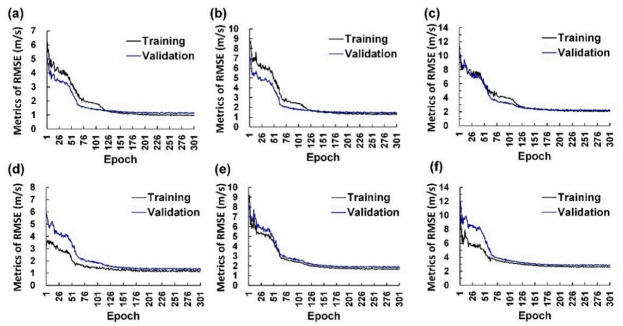
Learning curves of the training stage and validation stage using the optimal parameters combination of WIND-2_GRU for Longdong Buoy: (**a**) *t* + 1, (**b**) *t* + 3, and (**c**) *t* + 6 and for Liuqiu Buoy: (**d**) *t* + 1, (**e**) *t* + 3, and (**f**) *t* + 6.

**Figure A2:**
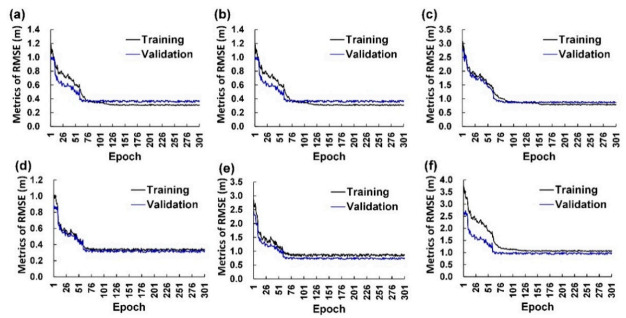
Learning curves of the training stage and validation stage using the optimal parameters combination of WAVE-4_GRU for Longdong Buoy: (**a**) *t* + 1, (**b**) *t* + 3, and (**c**) *t* + 6 and for Liuqiu Buoy: (**d**) *t* + 1, (**e**) *t* + 3, and (**f**) *t* + 6.

Figure A1 and Figure A2 in Appendix A depict the learning curves of the training stage and validation stage in the final optimal models for a lead time = 1 h, 3 h, and 6 h at both buoys. Figure A1 and Figure A2 show that the RMSE decreased as the epoch number increased using a training set. The training process is stopped after a certain number of epochs, or the metrics on the validation dataset increased obviously as compared to the metric at the prior epoch to avoid model overfitting. Since the results of Table 3, Table 4, Table 5 and Table 6 illustrate that WIND-based and WAVE using GRU layers exhibited superior RMSE performances to those using dense layers, this study determined to use the GRU layers as the model structures for learning the temporal dynamics of the time sequences of the inputs in the WIND-based and WAVE-based models.

## 4. Simulations

In this section, the testing set, i.e., Typhoons Usagi, Fung-wong, and Megi, were employed to simulate using the WIND-based and WAVE-based models and then evaluate the performance levels from various model outcomes.

### 4.1. Wind Prediction Outcomes

**Figure 8:**
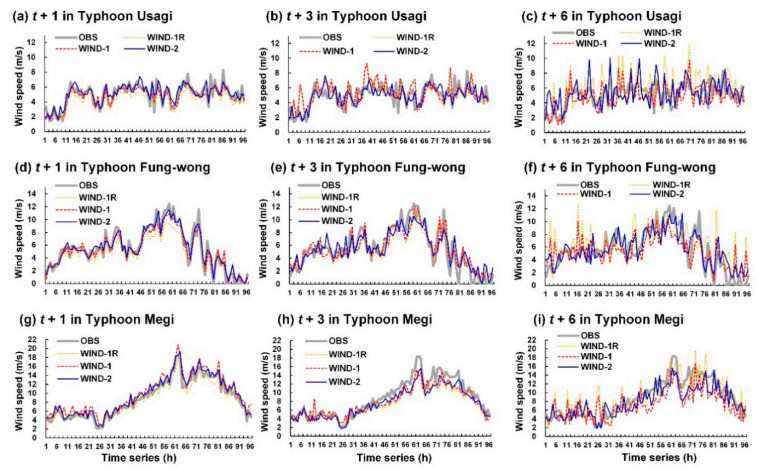
Simulation results of WIND-1R, WIND-1, and WIND-2 for the Longdong Buoy: (**a**–**c**) lead times of 1, 3, and 6 h for Typhoon Usagi, respectively; (**d**–**f**) lead times of 1, 3, and 6 h for Typhoon Fung-wong, respectively; (**g**–**i**) lead times of 1, 3, and 6 h for Typhoon Megi, respectively.

**Figure 9:**
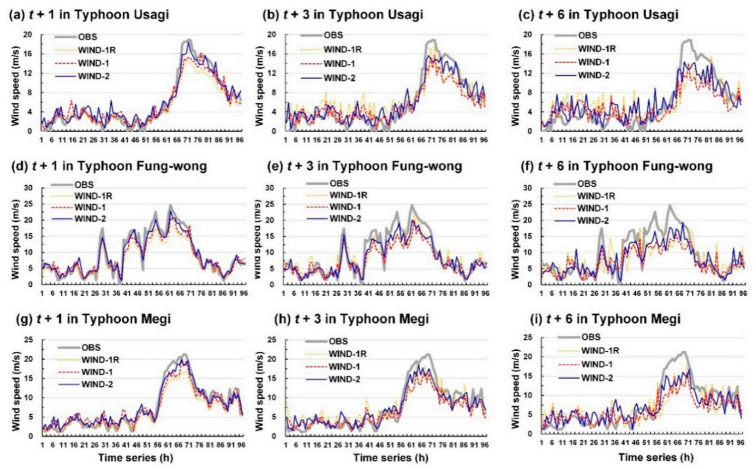
Simulation results of WIND-1R, WIND-1, and WIND-2 for Liuqiu Buoy: (**a**–**c**) lead times of 1, 3, and 6 h for Typhoon Usagi, respectively; (**d**–**f**) lead times of 1, 3, and 6 h for Typhoon Fung-wong, respectively; and (**g**–**i**) lead times of 1, 3, and 6 h for Typhoon Megi, respectively.

The wind velocities of the typhoons considered in this study were simulated to assess the developed wind velocity models. Figure 8 and Figure 9 display the wind speed prediction results for Longdong Buoy and Liuqiu Buoy, respectively.

Typhoon Usagi passed through the Luzon Strait without landing. The terrain did not interfere with this typhoon to a considerable extent; thus, the eye of the typhoon was still extremely obvious (Figure 6d). Consequently, a maximum wind speed of up to 18.9 m/s was observed at Liuqiu Buoy. The typhoon route of Fung-wong was near Liuqiu Buoy (Figure 6b), which caused high wind speeds at Liuqiu Buoy (up to 17.5 m/s). Then, it was northward along the eastern coast of Taiwan. Since Typhoon Fung-wong was possibly affected by terrain factors, it caused high wind speeds at Longdong Buoy (up to 12.5 m/s) lower than at Liuqiu Buoy. The center of Typhoon Megi passed through Central Taiwan. Thus, the influential radius of the typhoon circulation (approximately 250 km, Figure 6f) caused a maximum wind speed of 18.4 m/s at Longdong Buoy and 21.2 m/s Liuqiu Buoy.

The results depicted in Figure 8 and Figure 9 indicate that the simulated wind speed values obtained with the WIND-2 model were closer to the observation values than those obtained with the WIND-1R and WIND-1 models. Thus, WIND-2 is superior to WIND-1R, and WIND-1 determines the wind speed trend.

**Figure 10:**
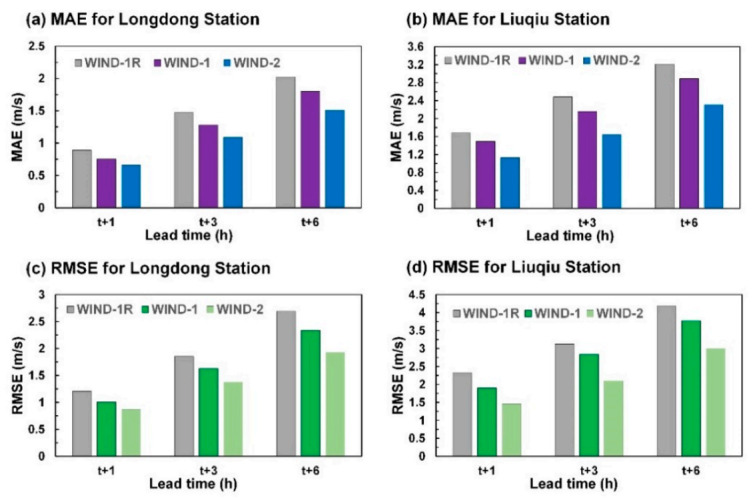
MAE and RMSE values for the wind velocity predictions: (**a**) MAE for Longdong Buoy, (**b**) MAE for Liuqiu Buoy, (**c**) RMSE for Longdong Buoy, and (**d**) RMSE for Liuqiu Buoy.

Figure 10 shows the mean absolute error (MAE) and RMSE for the predicted wind speeds using three test typhoons at Longdong Buoy and Liuqiu Buoy. The MAE is defined as follows:

(2) $$\mathrm{MAE}=\frac{1}{N_{k}}\sum_{i=1}^{N_{k}}\left|y_{k,i}^{\mathrm{obs}}-{\hat{y}}_{k,i}^{\mathrm{pre}}\right|$$

The MAE is an unbiased statistic for measuring the predictive capability of a model. The results indicated that the errors in the simulated wind speed values increased with an increase in the lead time from 1 to 6 h. The MAE and RMSE of the prediction values of WIND-2 were inferior to those of WIND-1R and WIND-1.

### 4.2. Wave Prediction Outcomes

**Figure 11:**
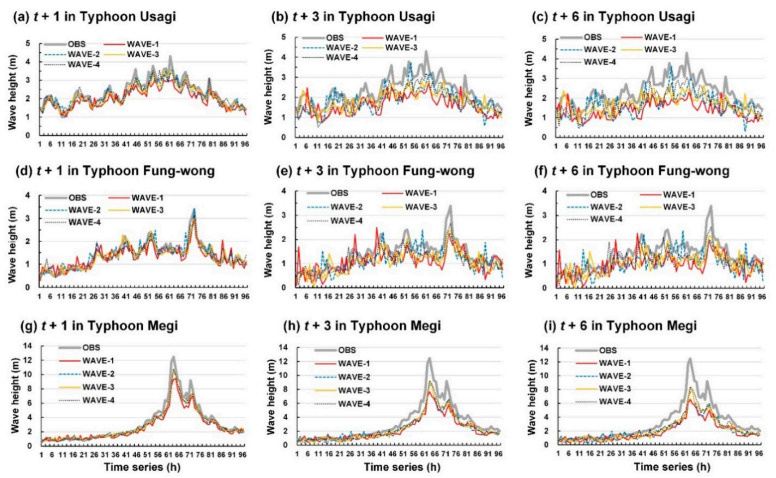
Results of WAVE-1, WAVE-2, WAVE-3, and WAVE-4 for Longdong Buoy: (**a**–**c**) lead times of 1, 3, and 6 h for Typhoon Usagi, respectively; (**d**–**f**) lead times of 1, 3, and 6 h for Typhoon Fung-wong, respectively; and (**g**–**i**) lead times of 1, 3, and 6 h for Typhoon Megi, respectively.

**Figure 12:**
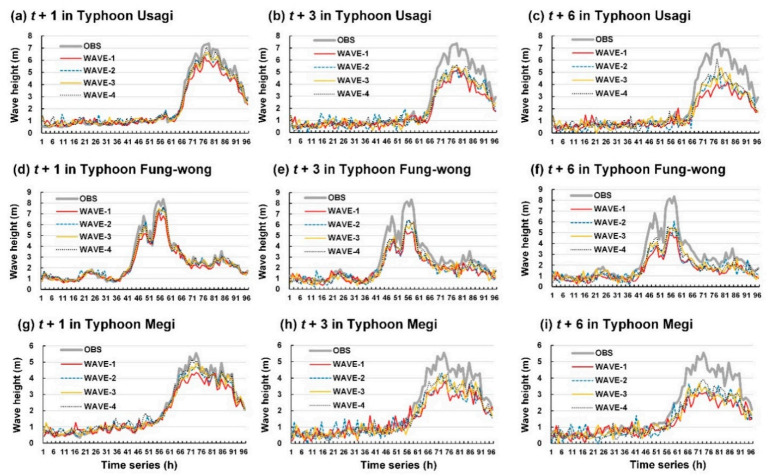
Results of WAVE-1, WAVE-2, WAVE-3, and WAVE-4 for Liuqiu Buoy: (**a**–**c**) lead times of 1, 3, and 6 h for Typhoon Usagi, respectively; (**d**–**f**) lead times of 1, 3, and 6 h for Typhoon Fung-wong, respectively; and (**g**–**i**) lead times of 1, 3, and 6 h for Typhoon Megi, respectively.

The wave height of the test typhoons was also simulated to assess the performance of the four wave height prediction models. Figure 11 and Figure 12 show the wave height prediction results for Longdong Buoy and Liuqiu Buoy, respectively. Typhoon Usagi passed through the Luzon Strait and caused a maximum wave height of 7.4 m at Liuqiu Buoy (Figure 12a). Typhoon Fung-wong was northward along the eastern coast of Taiwan and caused a maximum wave height of 3.4 m at Longdong Buoy (Figure 11d) and 8.4 m at Liuqiu Buoy (Figure 12d). In addition, Typhoon Megi passed through Central Taiwan and influenced almost all the regions of Taiwan. It caused a maximum wave height of 12.5 m at Longdong Buoy (Figure 11g) and 5.6 m at Liuqiu Buoy (Figure 12g).

**Figure 13:**
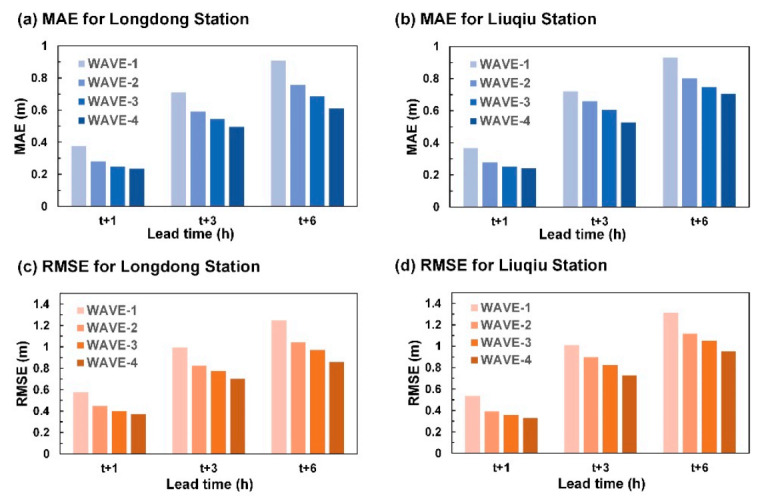
MAE and RMSE values for the wave height predictions: (**a**) MAE for Longdong Buoy, (**b**) MAE for Liuqiu Buoy, (**c**) RMSE for Longdong Buoy, and (**d**) RMSE for Liuqiu Buoy.

The results of this study indicated that WAVE-1 and WAVE-4 could determine the wave height trends at Longdong Buoy and Liuqiu Buoy for a lead time of 1 h. For lead times of 3 and 6 h, the four wave height prediction models exhibited different prediction efficacies. The prediction values of the WAVE-1 model exhibited high errors. The prediction values of the WAVE-2, WAVE-3, and WAVE-4 models were closer to the observation values than those of WAVE-1. Figure 13 displays the MAE and RMSE values of the predicted wave height results for the two buoys. The results indicated that the wave height simulation error increased for both buoys, with an increase in the lead time from 1 to 6 h. WAVE-1 exhibited the highest MAE and RMSE values, whereas WAVE-4 exhibited the smallest MAE and RMSE values.

### 4.3. Overall Performance for Predicting the Wind Velocity and Wave Height

The coefficient of efficiency (COE) and correlation coefficient (CORR) were used in this study to determine the overall prediction performance of the developed wind speed and wave height models. The COE and CORR are, respectively, expressed as follows:

(3) $$\mathrm{COE}=1-\frac{\sum_{i=1}^{N_{k}}{\left(y_{k,i}^{\mathrm{obs}}-{\hat{y}}_{k,i}^{\mathrm{pre}}\right)}^{2}}{\sum_{i=1}^{N_{k}}{\left(y_{k,i}^{\mathrm{obs}}-\bar{y^{\mathrm{obs}}}\right)}^{2}}$$

(4) $$\mathrm{CORR}=\frac{\sum_{i=1}^{N_{k}}\left(y_{k,i}^{\mathrm{obs}}-\bar{y^{\mathrm{obs}}}\right)\left({\hat{y}}_{k,i}^{\mathrm{pre}}-\bar{{\hat{y}}^{\mathrm{pre}}}\right)}{\sqrt{\sum_{i=1}^{N_{k}}{\left(y_{k,i}^{\mathrm{obs}}-\bar{y^{\mathrm{obs}}}\right)}^{2}\sum_{i=1}^{N_{k}}{\left({\hat{y}}_{k,i}^{\mathrm{pre}}-\bar{{\hat{y}}^{\mathrm{pre}}}\right)}^{2}}}$$

where $\bar{y^{\mathrm{obs}}}$ and $\bar{{\hat{y}}^{\mathrm{pre}}}$ are the means of all the observation values and prediction values, respectively. The larger COE and CORR values typically indicate favorable performance levels. The accurate model results should have COE and CORR values close to 1.

**Figure 14:**
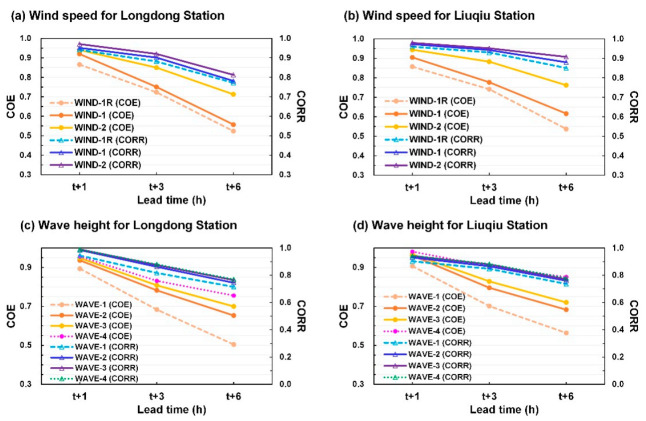
COE and CORR values of the wind speed predictions at (**a**) Longdong Buoy and (**b**) Liuqiu Buoy and those of the wave height predictions at (**c**) Longdong Buoy and (**d**) Liuqiu Buoy.

Figure 14 displays the COE and CORR results for the developed wind speed and wave height prediction models. The results indicated that (1) WIND-2 exhibited higher COE and CORR values than WIND-1R and WIND-1 for both buoys in the lead time from 1 to 6 h, and (2) WAVE-4 exhibited the highest COE and CORR values for both buoys, whereas WAVE-1 exhibited the lowest COE and CORR values in the lead time from 1 to 6 h.

### 4.4. Evaluation of the Classification for Wind Velocity and Wave Height

In this section, this study proceeded to calculate the predictive error for the classifications of the wind velocity and wave height. According to the wind and wave classifications provided by the CWB of Taiwan [62], winds can be classified into three categories: winds with a velocity less than 8.0 m/s were low winds, those with a velocity between 8.0 m/s and 10.7 m/s were moderate winds, and those with a velocity higher than 10.7 m/s were high winds. Then, the waves were classified into the three categories: waves with a height less than 1.5 m were low waves, those with a height between 1.5 m and 2.5 m were moderate waves, and those with a height higher than 2.5 m were high waves. To further analyze the different wind and wave strengths, the criterion BIAS was estimated using

(5) $$\mathrm{BIAS}=\frac{1}{N_{k}}\sum_{i=1}^{N_{k}}y_{k,i}^{\mathrm{obs}}-{\hat{y}}_{k,i}^{\mathrm{pre}}$$

**Figure 15:**
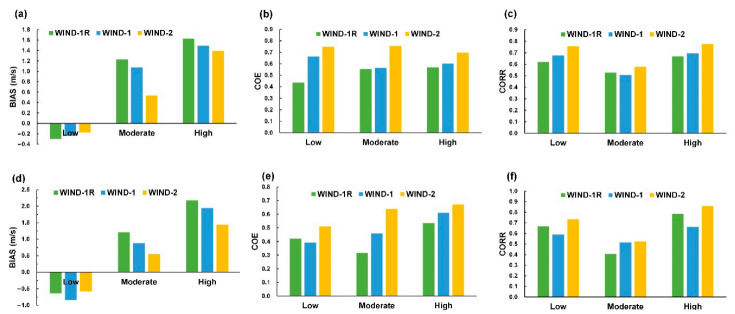
Metrics of the wind speed classifications at Longdong Buoy: (**a**) BIAS, (**b**) COE, and (**c**) CORR and those at Liuqiu Buoy: (**d**) BIAS, (**e**) COE, and (**f**) CORR.

**Figure 16:**
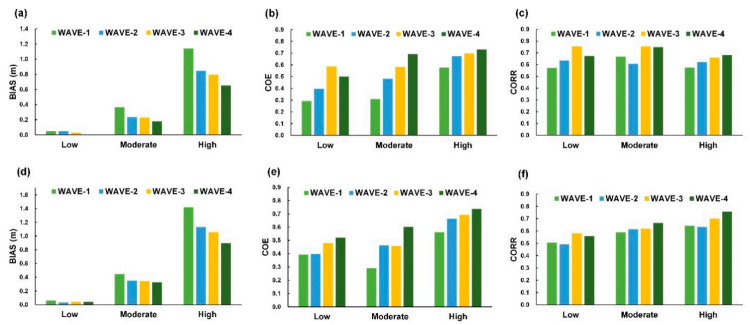
Metrics of the wave height classifications at Longdong Buoy: (**a**) BIAS, (**b**) COE, and (**c**) CORR and those at Liuqiu Buoy: (**d**) BIAS, (**e**) COE, and (**f**) CORR.

When the observation is greater than the forecast, the BIAS value is positive, indicating under-forecasting. The inverse, resulting in a negative BIAS, indicates over-forecasting. As shown in Figure 15 and Figure 16, the different wind and wave strengths were evaluated by calculating the average BIAS, COE, and COREE scores of the lead times = 1, 3, and 6 h for the model cases using three test typhoons at Longdong Buoy and Liuqiu Buoy. The results indicated the following.

For the wind velocity classifications, the BIAS scores (Figure 15a,d) revealed the predicted wind velocity overestimated in the low level for WIND-1R, WIND-1, and WIND-2 at both buoys and underestimated in the moderate and high levels. The greater bias is at the high level compared to those at the low and moderate levels, which means models underestimate at higher wind speeds. For the COE and CORR scores (Figure 15b,c,e,f), WIND-2 yields higher COE and CORR scores compared to WIND-1R and WIND-1 for all categories. Comparing the three metrics of the model cases, WIND-2 exhibits satisfactory results in the three categories of wind velocity. Furthermore, for the wave height classifications, the BIAS values for all the cases were positive values, which indicated that the wave heights were underdetermined at the three categories. According to Figure 16b,e, the results of the COE for both buoys exhibited a relatively low COE for small wave heights and relatively high for moderate and high levels. Moreover, comparing all the model cases, WAVE-4 provides promising results regarding the three categories of wave heights.

### 4.5. Discussion

The developed wind speed prediction models were WIND-1 and WIND-2. Data from the ground stations and buoys were used as the input data for WIND-1. Moreover, data from the ground stations and buoys, as well as radar reflectivity images, were used as the input data for WIND-2. The results indicated that the efficiency of WIND-2 was, respectively, 13.01% and 9.17% higher than those of WIND-1R and WIND-1 for Longdong Buoy. Furthermore, the efficiency of WIND-2 was, respectively, 15.11% and 9.72% higher than those of WIND-1R and WIND-1 for Liuqiu Buoy. Overall, WIND-2 exhibited a superior prediction performance to WIND-1R and WIND-1. WIND-2 had a higher efficiency than WIND-1R and WIND-1, because the combination of RNNs and CNNs of WIND-2 could extract wind field features from the radar reflectivity images more effectively than those of WIND-1R and WIND-1 could.

WAVE-1 was used as the benchmark model. The results indicated that the efficiency of WAVE-4 was 15.36% higher than that of WAVE-1 for Longdong Buoy. The COE efficiencies of WAVE-3 and WAVE-2 were 12.50% and 9.69% higher than that of WAVE-1 for Longdong Buoy, respectively. The efficiency of WAVE-4 was 15.10% higher than that of WAVE-1 for Liuqiu Buoy. Moreover, the efficiencies of WAVE-3 and WAVE-2 were 11.48% and 8.92% higher, respectively, than that of WAVE-1 for Liuqiu Buoy. Overall, the increased efficiencies of WAVE-2 and WAVE-3 were contributed to by the wind speed predictions of WIND-1 and WIND-2, respectively. However, only the wind speed information of single points (the buoys) was used. Thus, the efficiency of the WAVE-1–WAVE-3 models was lower than that of WAVE-4, which directly used radar reflectivity images. These images can provide additional information on spatial changes.

The prediction results obtained for Longdong Buoy and Liuqiu Buoy were compared. The COE efficiency of the WIND-2 model was 2.9% higher for Liuqiu Buoy than for Longdong Buoy. The efficiency of the WAVE-4 model was 2.7% higher for Liuqiu Buoy than for Longdong Buoy. The speculated reason for the results is that Longdong Buoy is influenced by the seabed topography. The water is shallower at Longdong Buoy (30 m) than at Liuqiu Buoy (78 m), whereas Longdong Buoy is closer to the land than Liuqiu Buoy. Moreover, the influence of the topography makes it difficult to predict the wind and wind-induced waves. This might be due to the mountain-induced flow deflections being mainly confined to the lower-level vortex, and the typhoon’s passage induced a mean cyclonic circulation pattern around the terrain of the Central Mountain Range of Taiwan [27]. In other words, the steep and high terrain causes typhoon track deflections and structure modifications. Thus, the typhoon route and land topography may have influenced the wind velocity and wave height predictions for the two buoys. The winds and waves at a buoy are stronger if the buoy is located at the windward side of the typhoon circulation than if the buoy is located at the leeward side.

**Figure 17:**
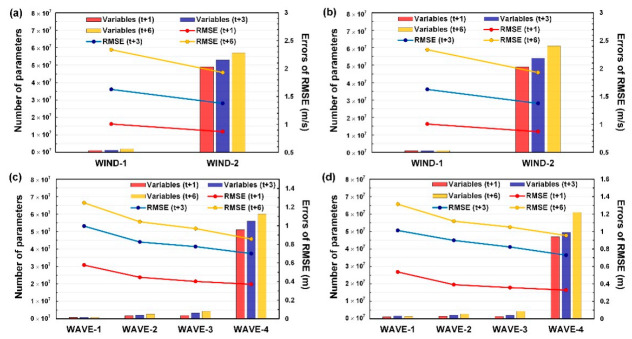
Computation cost among the model cases for WIND-based models at (**a**) Longdong Buoy and (**b**) Liuqiu Buoy and WAVE-based models at (**c**) Longdong Buoy and (**d**) Liuqiu Buoy.

In order to understand the computational cost of each model during the training phase, Figure 17 displays the relationships among the RMSE prediction errors, the total numbers of trainable variables, and the WIND-based and WAVE-based model cases. The figure indicates that the prediction errors decrease in the model cases of WIND-2 (Figure 17a,b) and WAVE-4 (Figure 17c,d) substantially, whereas the numbers of trainable variables of WIND-2 and WAVE-4 increased considerably. This is because the increased hyperparameters were used to extract the spatial and temporal features from the wind and wave data when the convolutional layers and GRU layers are conducted in modeling. Although WIND-2 and WAVE-4 produced superior prediction results compared to the other models (i.e., WIND-1, WAVE-1, WAVE-2, and WAVE-3), they required more computation costs for identifying the best set of hyperparameters in the learning phase during the model constructions.

## 5. Conclusions and Suggestion

In this study, real-time wind and wave changes in coastal waters during typhoon periods were predicted. The obtained wind and wave information can be used to prevent damage to infrastructures in international ports. This study used the ground station data, buoy data, and hourly radar reflectivity images collected by CWB ground stations. RNN-based GRUs and CNNs were combined and used to construct wind speed models. Then, the wind speed prediction results of the wind speed models were used to construct wave height prediction models. In this study, the data of 21 typhoons that affected Taiwan between 2013 and 2019 were collected. The wind speed and significant wave height in the coastal waters of the Keelung and Kaohsiung Ports over the lead times of 1–6 h were predicted and analyzed. The data of three test typhoons were analyzed: Typhoons Usagi, Fung-wong, and Megi.

The results indicated that, first, WIND-2 has a superior wind speed prediction performance compared to WIND-1, where WIND-2 can be used to identify the temporal and spatial changes in wind speeds using ground station data and reflectivity images. Second, WAVE-4 has the optimal wave height prediction performance, followed by WAVE-3, WAVE-2, and WAVE-1. The results of WAVE-4 revealed using the designed models using these data directly yielded optimal predictions of wind-based wave heights. Overall, the results indicated that the designed models could solve problems regarding typhoon-induced winds and waves prediction with high accuracy, because the combination of CNNs and GRUs is able to extract the features by convolutional operations and capture time dependencies from these time sequence data.

The recommendations of this study are as follows. First, because most of current methods for the predictions of wind and waves use numerical model (e.g., WRF and WAVEWATCH series), those outcomes from numerical models could be compared and considered as inputs used in our presented models. The influence of wind fields and terrain effects could be considered for more detail. For example, Wei [21] used the National Center for Environmental Prediction final reanalysis data on 1-degree by 1-degree resolution as the initial field and boundary conditions for simulating a WRF model and suggested that the interpolation method could be used to obtain the spatiotemporal sequences of the wind field effectively. Second, as mentioned earlier, this work collected radar reflectivity images with a sample time of one hour. However, the 6-min volume scanning radar data was recommended to be used in the training network for increasing the number of samples. It should be noticed that, when using a sample time of 6 min, the data of the ground station and buoy should be processed by interpolation into six time frames within 1 h.

Third, different remote-sensing images are recommended to be used in the modeling process. The parameters of radar reflectivity are associated with various acquisition limitations; for example, the radar coverage is limited due to terrain complexity and the shading problem [63]. Remote-sensing satellite images are recommended to be used in wind and wave prediction. It should be noticed that the wind from satellites in which the signals are observed from the clouds on top. Thus, the radar reflectivity signals are observed from above the ground to the middle level of the clouds, which is quite different.

Fourth, although the radar reflectivity images were used, the radar reflectivity image, which is a quantified representation of the reflectivity data, accuracy is lower than the original radar-based data (including reflectivity, radial velocity, and spectral width). Thus, the original radar-based data are recommended to be used as model inputs and increase the achievement of the favorable prediction efficiency.

## Figures and Tables

**Table 1:** Typhoons that occurred in the study area.

Typhoon	Periods	Intensity	Typhoon	Periods	Intensity
Soulik	11–13 July 2013	Severe	Nepartak	6–9 July 2016	Severe
Trami	20–22 August 2013	Mild	Meranti	12–15 September 2016	Severe
Kong-rey	27–29 August 2013	Mild	Malakas	15–18 September 2016	Moderate
Usagi	19–22 September 2013	Severe	Megi	25–28 September 2016	Moderate
Fitow	4–7 October 2013	Moderate	Nesat	28–30 July 2017	Moderate
Hagibis	14–15 June 2014	Mild	Haitang	30–31 July 2017	Mild
Matmo	21–23 July 2014	Moderate	Hato	20–22 August 2017	Moderate
Fung-wong	19–22 September 2014	Mild	Guchol	6–7 September 2017	Mild
Noul	10–11 May 2015	Severe	Talim	12–14 September 2017	Moderate
Chan-hom	9–11 July 2015	Moderate	Mitag	29 September–1 October 2019	Moderate
Linfa	6–9 July 2015	Mild			

**Table 2:** Designed cases with their corresponding skills and data and related referred studies.

Model Case	Algorithm	Input Data	Referred Paper
WIND-1	MLP/GRU	1D in-situ {**G**,**V**}	[21,23,29]
WIND-2	CNN + MLP/GRU	1D in-situ {**G**,**V**} and 2D image {**I**}	Present
WAVE-1	MLP/GRU	1D in-situ {**G**,**H**}	[32]
WAVE-2	MLP/GRU	1D in-situ {**G**,**H**} and predicted {**V**’} from WIND-1	[32]
WAVE-3	MLP/GRU	1D in-situ {**G**,**H**} and predicted {**V**’} from WIND-2	Present
WAVE-4	CNN + MLP/GRU	1D in-situ {**G**,**H**} and 2D image {**I**}	Present

**Table 3:** Calibration results of the WIND-1 model for the number of dense/GRU layers and the number of neurons in a dense/GRU layer at Longdong Buoy and Liuqiu Buoy.

Model	Buoy	Lead Time (h)					
		*t* + 1		*t* + 3		*t* + 6	
Parameters with Metrics		Layers, Neurons	RMSE (m/s)	Layers, Neurons	RMSE (m/s)	Layers, Neurons	RMSE (m/s)
WIND-1_dense	Longdong	1, 100	1.417	2, 130	2.018	2, 140	2.812
	Liuqiu	1, 70	1.763	2, 80	2.735	2, 100	3.884
WIND-1_GRU	Longdong	1, 140	1.315	1, 150	1.803	2, 150	2.570
	Liuqiu	1, 80	1.631	1, 90	2.576	1, 110	3.532

**Table 4:** Calibration results of the WIND-2 model for the structure types of the VGGNets, cropped image sizes, the number of GRU layers, and the number of neurons in a dense/GRU layer at Longdong Buoy and Liuqiu Buoy.

Model	Buoy	Lead Time (h)								
			*t* + 1			*t* + 3			*t* + 6	
Parameters with Metrics		Structure, Size	Layers, Neurons	RMSE (m/s)	Structure, Size	Layers, Neurons	RMSE (m/s)	Structure, Size	Layers, Neurons	RMSE (m/s)
WIND-2_dense	Longdong	VGG16, 96	3, 2300	1.312	CNN5_3, 96	3, 2600	1.401	CNN5_4, 128	4, 2500	2.473
	Liuqiu	VGG16, 128	3, 1800	1.327	CNN5_3, 128	4, 2000	1.869	CNN5_3, 128	4, 2400	3.013
WIND-2_GRU	Longdong	CNN5_3, 96	3, 2600	1.103	CNN5_3, 96	3, 2700	1.384	CNN5_4, 128	3, 3000	2.112
	Liuqiu	CNN5_3, 128	3, 2400	1.292	CNN5_3, 128	3, 2500	1.801	CNN5_4, 128	4, 2100	2.786

**Table 5:** Calibration results of the WAVE-1, WAVE-2, and WAVE-3 models for the number of dense/GRU layers and the number of neurons in a dense/GRU layer at Longdong Buoy and Liuqiu Buoy.

Model	Buoy	Lead Time (h)					
		*t* + 1		*t* + 3		*t* + 6	
Parameters with Metrics		Layers, Neurons	RMSE (m)	Layers, Neurons	RMSE (m)	Layers, Neurons	RMSE (m)
WAVE-1_dense	Longdong	2, 80	0.594	3, 100	0.961	3, 80	1.301
	Liuqiu	1, 60	0.481	3, 110	0.868	3, 110	1.418
WAVE-1_GRU	Longdong	1, 90	0.573	2, 130	0.903	2, 100	1.187
	Liuqiu	1, 60	0.443	2, 90	0.842	2, 120	1.202
WAVE-2_dense	Longdong	2, 100	0.456	2, 120	0.831	4, 100	0.991
	Liuqiu	2, 60	0.368	3, 110	0.887	3, 120	1.238
WAVE-2_GRU	Longdong	1, 110	0.447	2, 90	0.802	2, 100	0.902
	Liuqiu	1, 70	0.350	2, 100	0.868	2, 110	1.021
WAVE-3_dense	Longdong	2, 120	0.433	3, 90	0.782	4, 120	0.955
	Liuqiu	2, 100	0.372	3, 100	0.822	4, 110	0.979
WAVE-3_GRU	Longdong	2, 100	0.401	3, 100	0.748	3, 170	0.928
	Liuqiu	1, 120	0.348	2, 110	0.786	3, 180	0.948

**Table 6:** Calibration results of the WAVE-4 model for the structure types of the VGGNets, cropped image sizes, the number of dense/GRU layers, and the number of neurons in a dense/GRU layer at Longdong Buoy and Liuqiu Buoy.

Model	Buoy	Lead Time (h)								
			*t* + 1			*t* + 3			*t* + 6	
Parameters with Metrics		Structure, Size	Layers, Neurons	RMSE (m)	Structure, Size	Layers, Neurons	RMSE (m)	Structure, Size	Layers, Neurons	RMSE (m)
WAVE-4_dense	Longdong	CNN5_3, 96	2, 2200	0.358	CNN5_4, 96	4, 1500	0.746	CNN5_4, 128	4, 1600	0.876
	Liuqiu	VGG16, 96	2, 1900	0.321	CNN5_3, 128	3, 1900	0.723	CNN5_4, 128	4, 1500	0.941
WAVE-4_GRU	Longdong	CNN5_4, 96	3, 2100	0.347	CNN5_3, 128	4, 1400	0.728	CNN5_4, 128	4, 1700	0.844
	Liuqiu	CNN5_4, 128	2, 2300	0.309	CNN5_3, 128	3, 2100	0.705	CNN5_4, 128	4, 1800	0.919

## Data Availability

The related data were provided by Taiwan’s Central Weather Bureau, which are available at https://rdc28.cwb.gov.tw/ (accessed on 1 July 2020).
